# Decoding Polymer Architecture Effect on Ion Clustering, Chain Dynamics, and Ionic Conductivity in Polymer Electrolytes

**DOI:** 10.1021/acsaem.3c00310

**Published:** 2023-03-23

**Authors:** Recep Bakar, Saeid Darvishi, Umut Aydemir, Ugur Yahsi, Cumali Tav, Yusuf Ziya Menceloglu, Erkan Senses

**Affiliations:** † Department of Material Science and Engineering, Koç University, Sariyer, Istanbul 34450, Türkiye; ‡ Department of Chemical and Biological Engineering, Koç University, Sariyer, Istanbul 34450, Türkiye; § Department of Chemistry, Koç University, Sariyer, Istanbul 34450, Türkiye; ∥ Koc University Boron and Advanced Materials Application and Research Center (KUBAM), Sariyer, Istanbul 34450, Türkiye; ⊥ Department of Physics, Faculty of Science, Marmara University, Kadikoy, Istanbul 34722, Türkiye; # Faculty of Engineering and Natural Sciences, Sabanci University, Tuzla, Istanbul 34956, Türkiye; ∇ Koç University Surface Science and Technology Center (KUYTAM), Rumelifeneri yolu, Sariyer, Istanbul 34450, Türkiye

**Keywords:** homopolymer electrolytes, poly(ethylene oxide), polymer architecture, ionic conductivity, free volume, viscosity, phase diagram, ion pairing and clustering

## Abstract

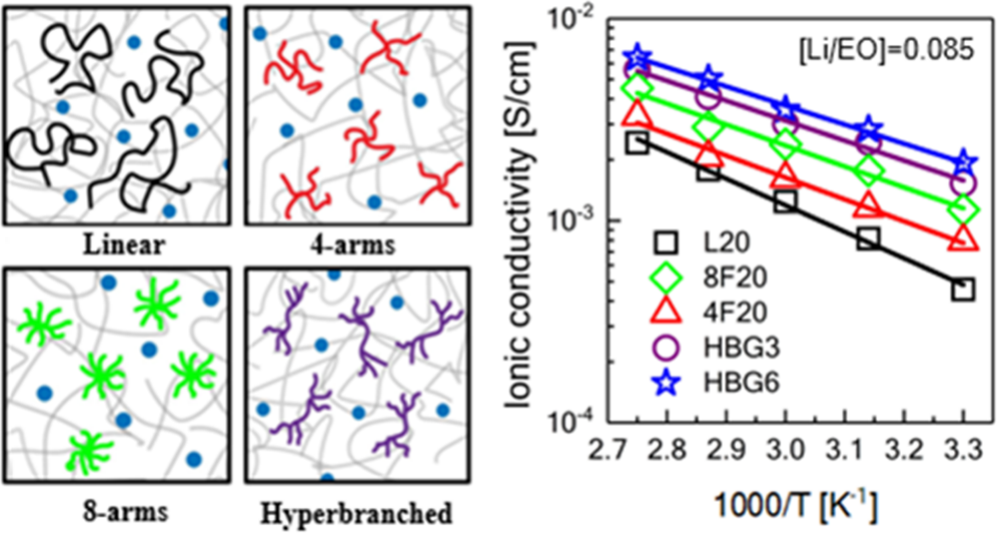

Poly(ethylene oxide) (PEO)-based polymer electrolytes are a promising class of materials for use in lithium-ion batteries due to their high ionic conductivity and flexibility. In this study, the effects of polymer architecture including linear, star, and hyperbranched and salt (lithiumbis(trifluoromethanesulfonyl)imide (LiTFSI)) concentration on the glass transition (*T*_g_), microstructure, phase diagram, free volume, and bulk viscosity, all of which play a significant role in determining the ionic conductivity of the electrolyte, have been systematically studied for PEO-based polymer electrolytes. The branching of PEO widens the liquid phase toward lower salt concentrations, suggesting decreased crystallization and improved ion coordination. At high salt loadings, ion clustering is common for all electrolytes, yet the cluster size and distribution appear to be strongly architecture-dependent. Also, the ionic conductivity is maximized at a salt concentration of [Li/EO ≈ 0.085] for all architectures, and the highly branched polymers displayed as much as three times higher ionic conductivity (with respect to the linear analogue) for the same total molar mass. The architecture-dependent ionic conductivity is attributed to the enhanced free volume measured by positron annihilation lifetime spectroscopy. Interestingly, despite the strong architecture dependence of ionic conductivity, the salt addition in the highly branched architectures results in accelerated yet similar monomeric friction coefficients for these polymers, offering significant potential toward decoupling of conductivity from segmental dynamics of polymer electrolytes, leading to outstanding battery performance.

## Introduction

Lithium-ion rechargeable batteries have been extensively used in different applications, including electric vehicles, electronics, and energy storage systems.^1−5^ Currently, these batteries utilize liquid electrolytes associated with disadvantages and potential risks, which could lead to explosions, leakages, fires, and environmental hazards.^1,5−8^ To overcome these problems, polymer electrolytes (PEs) have been widely investigated as replacements for liquid electrolytes in lithium-ion batteries.^1,6,9,10^ Despite offering significant advantages such as high flexibility, decent mechanical stability, facile processability, large electrochemical window, and superior energy density,^4,6,8,10,11^ their low ionic conductivity at room temperature (<10^–5^ S/cm)^12,13^ still remains a major challenge for large-scale commercialization.

Various polymers, including poly(acrylonitrile), poly(vinylidene fluoride) (PVDF), and poly(methyl methacrylate), have been studied as solid polymer electrolytes (SPEs);^6^ the most preferred one is still poly(ethylene oxide) (PEO) due to its fast segmental dynamics in the amorphous state,^13^ low glass-transition temperature (*T*_g_ ∼ 220 K^13^), good electrochemical stability, as well as its ability to dissolve different types of lithium salts.^1,6,9,10^ Therefore, below its melting temperature (*T*_m_ = 60 °C, in the neat form),^13,14^ PEO has low ionic conductivity ranging between 10^–6^ and 10^–8^ S/cm, whereas it rapidly increases above the melting temperature at the cost of severe loss of its mechanical strength upon melting of the crystals, which otherwise reinforce the material.^15^ Ion conduction primarily occurs in the amorphous phase where the ion motion is facilitated by segmental dynamics of the polymer.^10,13,15,16^ Majority of the previous studies, therefore, aimed for accelerated relaxation of polymer segments without much sacrificing of mechanical strength. This is an extremely challenging task since the fast segmental motion results in reduced viscoelasticity of the linear polymers that are commonly employed; mechanisms to decouple ionic conductivity from nanoscale monomeric motions are urgently needed.

Several methods such as employing architectural changes and various chain lengths in PEO-based SPEs have been applied by different studies with the objective of developing high Li^+^-ion conductivity.^1,6,10,13,15,17,18^ Increasing branching would lower the friction by diffusing polymer chains with a smaller molecular weight, resulting in low viscosities due to the lack of entanglements in the polymer architecture when nonlinear topologies are used in preparing these SPEs.^6,7,13,19−21^ For example, Butzelaar et al.^10^ and Lee et al.^22^ developed PEO-based electrolytes using star PEO topologies with a constant number of arms at the constant LiTFSI salt concentration and found significantly enhanced room-temperature ionic conductivity attributed to suppressed crystallization by the nonlinear architecture. In a similar study, Marzantowicz and co-workers,^18^ using a star-branched poly (ethylene oxide) with nearly 20 arms over a limited range of LiTFSI salt concentration, reported ionic conductivity values comparable to linear PEO-based electrolytes, which reached its maximum at the molar ratio of [EO/Li = 10]. The ionic conductivity declined with further lithium addition because of possible ion clustering formation, suggesting highly dependent ionic conductivity with salt concentration. Furthermore, Lee et al.^1^ and Chen et al.^6^ employed hyperbranched poly (ethylene oxide) with a fixed LiTFSI salt loading. They reported enhanced Li-ion conductivity with sufficient mechanical stability mainly because of efficiently suppressed crystallization, faster segmental motion, and decreasing viscosity owing to nanoconfinement effects and end group modification. In another work studying the PEO/LiTFSI system with a fixed salt concentration, Thelakkat et al.^23^ investigated highly branched copolymers having PEO side chains and a rigid polymer forming the backbone by correlating the ionic conductivity with changing polymer stiffness and found that higher ionic conductivity was achieved with decreasing chain stiffness. Devaux and co-workers^7^ reported decreasing the chain length reduced the viscosity with the incremental contribution of the end groups in increasing the free volume, which eventually resulted in higher ionic transportation. While these previous findings show separate evidence that the macromolecular shape leading to different polymers as well as number of free chain ends related to functionality (branching) can play a significant role, there is no systematic and comprehensive study investigating how the polymer architecture of the polymer influences the phase behavior, segmental dynamics, rheology, and ionic conductivity in PEO electrolytes.

Very recently, in our study,^13^ we investigated the effect of different PEO topologies (linear, stars, hyperbranched, and bottle brush) on ionic conductivity when PEO is blended with a linear PMMA matrix more at a constant lithium salt concentration of LiTFSI [Li/EO] = 0.085. We found that higher ionic conductivities can be achieved for the electrolytes including nonlinear PEO architectures with moderate branching when compared to their linear counterparts. This is mainly because of the increasing miscibility limit of PEO toward higher PEO/PMMA fractions and, more importantly, the faster segmental dynamics of PEO that is decoupled from slow/glassy PMMA. Still, it is unclear whether ion clustering and electrochemical properties of the homopolymer electrolytes are architecture-dependent and can be correlated with the changes in free volume and microscopic relaxation rate. Therefore, the phase and conductivity diagrams of PEO along with free volume characteristics, rheological properties, and segmental dynamics over a wide range of LiTFSI salt concentrations need to be systematically investigated in detail.

**Figure 1:**
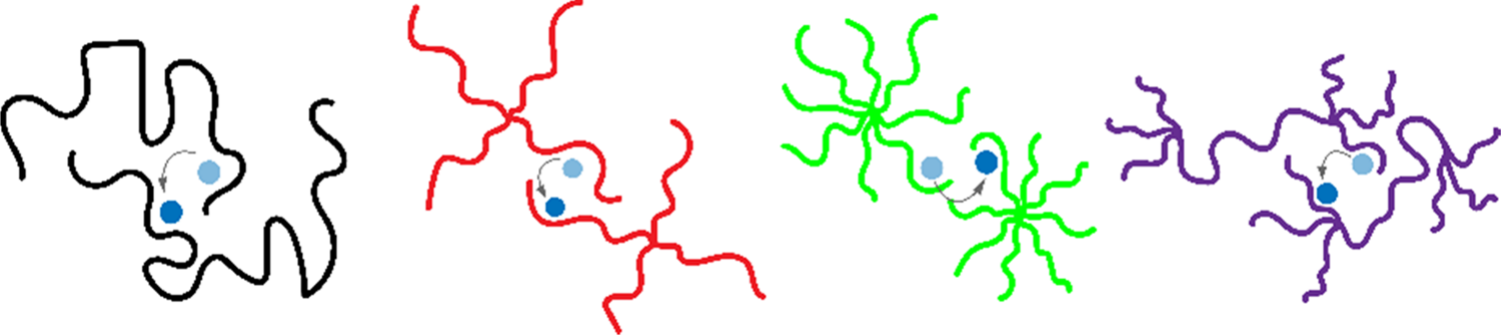
Schematic representation of the PEO-based solid polymer electrolytes with the linear, four-arm star, eight-arm star, and hyperbranched used in this study Blue filled circles represent the Li^+^ ion, and arrows show the interchain ion hopping of the ions.

Herein, we utilized various PEO topologies with hydroxyl end groups including linear, stars (four arms, eight arms), and hyperbranched PEO as shown in Figure 1. Our results show that the phase boundaries depend on PEO architectures, forming completely amorphous mixtures at lower salt loadings with increasing branching. The salt concentration-dependent ionic conductivity trend is similar for all PEO topologies, reaching maxima around [Li/EO = 0.085], followed by a dramatic decrease due to ion pairing and clustering effects. The comparison at the fixed salt loading, [Li/EO = 0.085], reveals that the ionic conductivity is monotonically enhanced by the degree of branching such that the electrolyte with hyperbranched PEOs yielded as much as three times higher ionic conductivity in the liquid state. This behavior is attributed to the excess free volume provided by free ends of the branched polymer architectures, which is verified by positron annihilation lifetime spectroscopy. Interestingly, quasielastic neutron backscattering results on electrolytes of PEO with the same total molar mass show that the effect of compactness, which reduced dynamics in the four-arm star, is overcome by the enhanced mobility of the free ends provided by a higher functionality polymer (eight-arm star and hyperbranched ones). This results in a decrease of the average monomeric friction coefficient in these samples by about 40% with respect to the linear PEO. The resulting fast dynamics is, however, independent of architecture; thus, the coupling between ion mobility and segmental dynamics reported for linear polymers is not as strong for nonlinear polymers.

## Experimental Methodology

### Materials

Linear PEO, hyperbranched (third and sixth generation) PEO, and lithium bis(trifluoromethane)sulfonamide, LiTFSI salt, were purchased from Sigma-Aldrich. Four-arm and eight-arm star PEOs were supplied by Creative PEGWorks. All polymers were used as received without modification. The polymer characteristics are displayed in Table 1. The chemical structure of the PEOs with hydroxyl end groups is provided in Figure S1.

**Table 1:** Molecular Characteristics including Functionality, Dispersity, Total, and Arm Molecular Weight of the PEO Samples Used in This Study

PEO architecture	short name	functionality/arm number (*f*)	total molecular weight (*M_n_*) [kg/mol]	arm molecular weight [kg/mol]	dispersity (*D*)
linear	L20	2	20	10	1.10
4-arms star	4F5	4	5	1.25	1.03
4-arms star	4F10	4	10	2.5	1.03
4-arms star	4F20	4	20	5	1.03
8-arms star	8F10	8	10	1.25	1.10
8-arms star	8F20	8	20	2.5	1.10
8-arms star	8F40	8	40	5	1.10
hyperbranched	HBG3	3rd generation	20	NA	<1.5
hyperbranched	HBG6	6th generation	35	NA	<1.5

### Preparation of Salt-Free Samples and Electrolytes

Salt-free samples for neat PEOs with linear (*M*_w_ ∼ 20 kDa), four-arm star (*M*_w_ ∼ 5, 10, 20 kDa), eight-arm star (*M*_w_ ∼ 10, 20, 40 kDa), HBG3 (*M*_w_ ∼ 20 kDa), and HBG6 (*M*_w_ ∼ 35 kDa) topologies were prepared by the solution casting technique. Polymers were first dissolved in acetonitrile (ACN) at 30 mg/mL. Then, the solutions were stirred with a magnetic stirrer at room temperature for 6 h. Later, the desired amount of lithium bis (trifluoromethane) sulfonamide, LiTFSI, was dissolved in acetonitrile in a glovebox filled with argon, and the salt solution was stirred with a magnetic stirrer at room temperature for 6 h. Next, the desired amount of salt solution was added into the polymer solutions in the glovebox, and they were continuously stirred for 48 h. The electrolyte solutions were then cast onto glass Petri dishes and evaporated slowly at room temperature overnight in a glovebox filled with argon and annealed at 100 °C in a vacuum oven for 48 h to ensure complete removal of the solvent.

### Characterization

#### X-ray Diffraction (XRD)

The XRD patterns of the pure PEO and the electrolytes were recorded using the Bruker D8 Phaser—X-ray diffractometer with a Cu Kα source. The samples were placed on the surface of a glass plate, which was heated to 90 °C by a thermoelectric module and waited until temperature stabilization was reached on the surface. Then, the diffraction data were obtained at this temperature with Bragg’s angles (2θ) varying from 5 to 60° at a rate of 0.1°.

#### Differential Scanning Calorimetry (DSC)

The differential scanning calorimetry (DSC) samples were prepared by putting ∼8–10 mg of material in aluminum pans provided by TA Instruments. DSC experiments of the neat PEOs and their electrolytes were carried out with a TA Instruments DSC Q25 instrument equipped with a refrigerated cooling system. An aluminum pan was used as a reference. To eliminate the temperature history completely, all of the samples were heated to 120 °C and waited for 5 min. Samples were then quickly cooled down to −85 °C at the rate of 20 °C/min. DSC scans were then collected at the rate of 10 °C/min during the heating process to 120 °C. Eventually, the step of the heat capacity in DSC measurements was used to estimate the glass-transition temperatures for the samples.

#### Fourier Transform Spectroscopy (FT-IR)

FT-IR characterization was performed on the pure PEOs and the electrolytes using the Thermo Scientific iS10 FT-IR spectrometer in the wavenumber range of 4000–600 cm^–1^ with 64 scans for each spectrum.

#### Dynamic Light Scattering

The DLS experiments were performed on a Zetasizer NanoZS (Malvern Instrument) operating in the backscattering mode at an angle of 173° and at a temperature of 25 °C using a dilute PEO/solvent (acetonitrile) solution containing a PEO concentration of 1 mg/mL.

#### Rheology

Rheological measurements in the melt state were performed using an Anton Paar (MCR 302) rheometer, equipped with a cone geometry (diameter 25 mm, and 1° angle), and at a constant temperature of 75 °C. The viscosity of the samples was investigated in a shear rate table mode (0.1–1000 rad/s).

#### Electrochemical Impedance Spectroscopy (EIS)

Impedance characterization was carried out using an Autolab Potentiostat Galvanostat PGSTAT (Metrohm, The Netherlands) in the two-electrode configuration for PEO-based electrolytes. This arrangement was used to investigate electrode properties in liquid-state systems. The measurement frequency varied between 1 Hz and 1 MHz. Each SPE blend disc was sandwiched between two stainless steel blocking electrodes under an argon atmosphere in a glovebox located at Koç University Boron and Advanced Materials Application and Research Center (KUBAM) and sealed in an MTI Split Cell to measure the complex impedance spectra. After waiting for temperature stabilization with a margin of 0.1 °C at the experimental temperature of interest, the experiments were also carried out at various temperatures between 30 and 90 °C. The data were analyzed to characterize real and imaginary impedances using NOVA software to form Nyquist plots used for estimating resistance of the samples and the ionic conductivity.

#### Positron Annihilation Lifetime Spectroscopy (PALS)

The purpose of PALS measurement is to determine the free volume behavior of PEOs with varying topologies (linear, four arms, eight arms, and HBG3) for the temperature range from 30 to 90 °C. To accomplish this, we employed a fast–fast coincidence system measuring the time interval between the prompt γ-ray of 1274 keV as the start signal and the annihilation γ-ray of 511 keV as the stop signal. About 30 μCi of ^22^NaCl source on a thin aluminum foil (5 μm thick) was inserted between two pieces of film samples with a thickness of 2 mm or more. For each γ-ray detection, a plastic scintillator (BC422, Saint-Gobain Crystals, Hiram, OH) was connected to photomultiplier tubes (PMT R2059, Hamamatsu Photonics Deutschland GmbH, Herrsching, Germany) mounted on a PMT base (265A, Ortec AMETEK GmbH, Meerbusch, Germany) operating at negative 2100 volts. Two constant fractional differential discriminators (CFDD 583B, Ortec AMETEK GmbH, Meerbusch, Germany) for window settings of 1274 and 511 keV and for timing signals were used. These were connected to a time-to-amplitude converter (TAC 266, Ortec AMETEK GmbH, Meerbusch, Germany) to convert pulses of different heights into a time-to-pulse-height signal. The converted signals were fed to a multichannel analyzer (MCA ASPEC-927, Ortec AMETEK GmbH, Meerbusch, Germany). The spectroscopic data obtained from MCA were analyzed using the LT polymer to obtain the lifetimes and intensities, providing facts on the free volume. The resolution of the system was about 350 ps using a Si crystal as a reference, and the source contribution was about 10.5%, with lifetime contributions of 0.2 ns and 0.4 ns and respective intensities of 80 and 20%.

#### Quasielastic Neutron Scattering (QENS)

QENS experiments were performed using a High-Flux backscattering spectrometer (HFBS) at the NIST Center for Neutron Research (NCNR) at 400 K and at the dynamic range of ± 11 μeV. The samples about ≈100 μm thick were spread onto aluminum sheets and rolled into an annular shape to fit Al containers. The samples were heated to 400 K and equilibrated for one hour before starting the measurements.

## Results and Discussion

### Polymer Phase Behavior in Electrolytes

**Figure 2:**
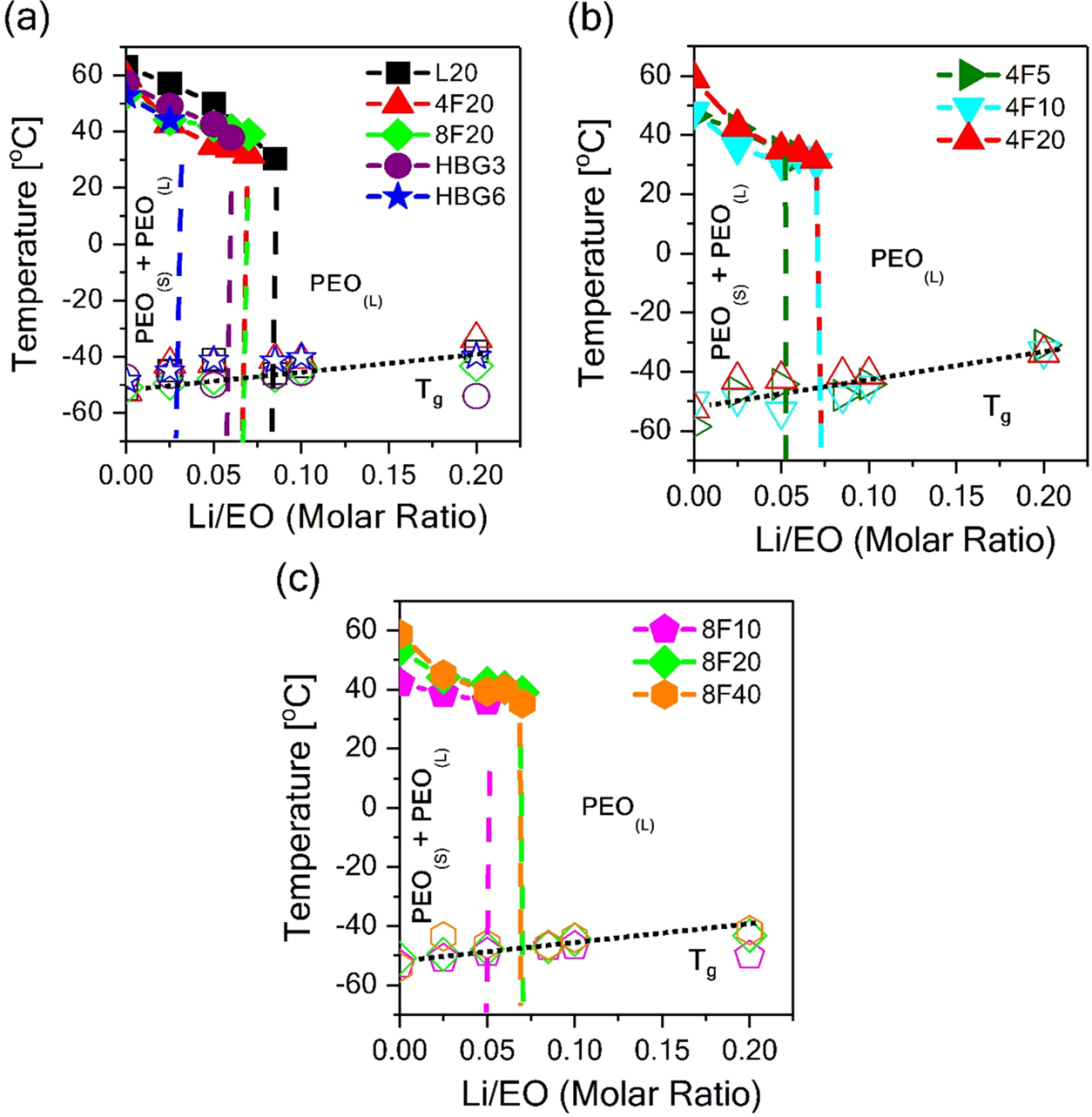
Phase diagrams of the PEO-LiTFSI systems with respect to different PEO architectures including linear, star, and hyperbranched topologies with (a) the same molecular weight (*M*_w_ ∼ 20 kDa), (b, c) with varying molecular weights (*M*_w_ ∼ 5, 10, 20 kDa) for four-arm and (*M*_w_ ∼ 10, 20, 40 kDa) eight-arm star topologies over a wide range of salt concentrations ([Li/EO] ranging from 0 to 0.2). The diagram was derived from a calorimetric analysis of the DSC data. The filled and unfilled squares represent melting and glass-transition temperatures, respectively.

In the literature, previous phase diagrams were constructed based on linear PEO;^24^ we hereby include various other PEO architectures systematically to explore the architectural influences on phase behavior. The crystallization, melting, and glass-transition temperatures of PEO/LiTFSI electrolytes were investigated using differential scanning calorimetry (DSC). Figure 2 shows the constructed phase diagrams based on the salt concentration ranging from 0 to 0.2 [Li/EO] molar ratios for linear, four-arm star and eight-arm star, and hyperbranched PEO with varying molecular weights. This phase diagram contains two distinctive PEO phases, specifically a completely liquid phase labeled as PEO_(l)_ where no crystalline PEO is present and the mixture of solid PEO_(s)_ and liquid PEO_(l)_ in which crystalline and amorphous PEO domains coexist. In addition, the measured glass-transition temperatures (*T*_g_s) at varying salt loadings are shown. The open symbols represent the glass-transition temperatures, whereas the filled symbols are used for melting points (*T*_m_s). The glass-transition and melting temperatures of the neat (salt-free) PEOs for various architectures ranged around −55 and 60 °C, respectively (the representative DSC thermographs for the melting and glass-transition behaviors are given in Figure S2; see the DSC results in the Supporting Information for the details of DSC thermographs obtained for all PEO topologies). No significant change in *T*_g_ is observed for the salt-free PEO, in agreement with the previous reports.^25−27^*T*_m_, however, was more architecture-dependent such that the nonlinear chain architectures usually display lower melting temperatures (with a shift in *T*_m_ as high as 20 K). Also, *T*_m_ decreases with decreasing arm molecular weight of star PEOs for the same functionality. This is likely due to the slower crystallization kinetics with decreasing arm length, resulting in thinning of the crystalline lamellae.^20,28,29^ It is also seen in Figure S2 that melting enthalpies for the neat nonlinear architectures were lower, suggesting less crystallization with a higher degree of branching when compared to the linear chains due to the restricted mobility of the densely packed monomers near the branch points.^20,21,28,30^ This is more apparent for linear, four-arm star, eight-arm star, and hyperbranched topologies at the same total molar mass of 20 kDa, where the degree of crystallinity (*X*_c_) is monotonically decreased from 74.9% for linear to 31.5% for hyperbranched PEO (HBG6).

Addition of salt gradually results in elevations of the *T*_g_ values, whereas it reduces the melting points in all electrolytes primarily because of ion–dipole interactions between EO and salt ions, which restricts the mobility of the polymer segments.^18,24,31−33^ As seen in Figure 2a, the incorporation of the lithium salt into the PEO matrix considerably affected the phase boundaries. The decrease in melting temperatures (*T*_m_) in electrolytes was architecture-dependent. More specifically, increasing branching at the same total molar mass of 20 kDa lowered the *T*_m_ more significantly, implying a higher degree of coordination between EO units and the salt ions as the degree of branching increased (see Table S1 values in the SI).^38^ Similarly, the addition of the LiTFSI salt into PEO matrices severely decreased the crystallization with decreasing melting enthalpies for all architectures, but the extent of the relative decrease was architecture-dependent such that the higher the degree of branching, the lower the enthalpies (for the same total molar mass).^13,20,28,29^ More importantly, the fully amorphous PEO_(l)_ phase boundary shifts toward lower salt concentrations with increasing branching. Specifically, highly branched PEO systems, namely, HBG3 and HBG6, become fully amorphous at Li/EO > 0.06 and Li/EO > 0.025, respectively, whereas four-arm and eight-arm star PEO electrolytes are in the fully liquid state above at Li/EO > 0.07. Note that the linear PEO/LiTFSI blend electrolyte retained the semicrystalline nature of PEO up to the molar ratio of Li/EO = 0.085. As the chemistry did not change between the polymer architectures, the ability of dissolving more LiTFSI homogeneously in the matrices is due to the uneven spatial arrangement of the monomers originating from their shape. Figure 2b,c compares the effect of arm length of four-arm and eight-arm star polymers. It is obvious that the stars with the shortest unentangling arms display an enhanced liquid phase at lower salt concentrations due to an excess number of chain ends in compact short-arm stars. This observation is important to enhance ionic conductivity in these systems, as we can eliminate crystallization in nonlinear topologies and increase *T*_g_ without much affecting the bulk viscosity. This decreases the possibility of the formation of ion clusters, which eventually helps us optimize salt concentration and facilitate ion transportation. Overall, all of these results indicate that the addition of LiTFSI salt into PEO matrices of different architectures can significantly alter the phase behavior of PEO electrolytes, which modifies ion dissolution and ion pairing, thus impacting the electrolyte characteristics.

### Ion–Polymer Interaction and Clustering

**Figure 3:**
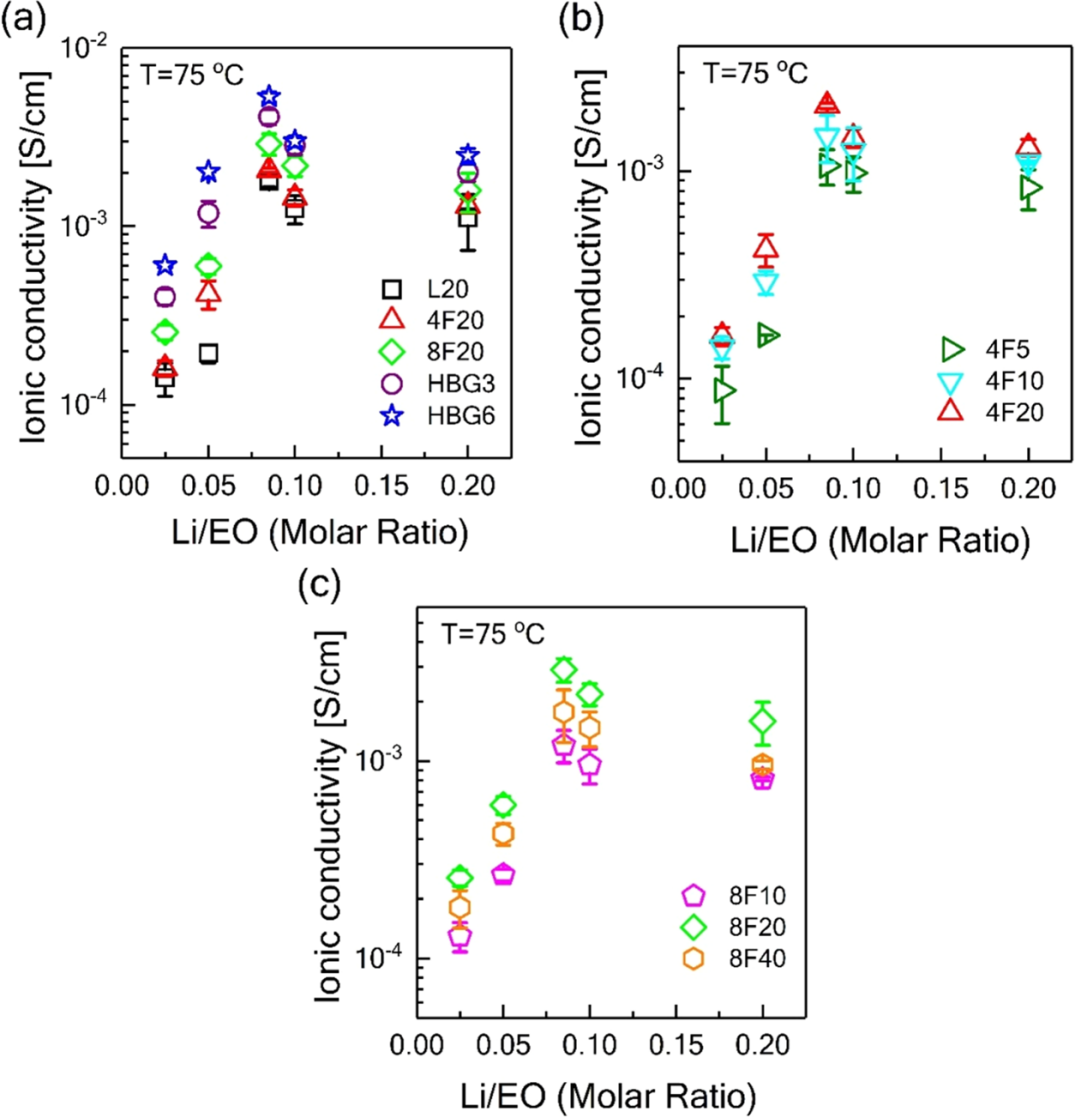
Ionic conductivity of PEO-based electrolytes at 75 °C with various architectures including linear, star, and hyperbranched with (a) the same molecular weight (*M*_w_ ∼ 20 kDa) and (b, c) with varying molecular weights (*M*_w_ ∼ 5, 10, 20 kDa) for four-arm and (*M*_w_ ∼ 10, 20, 40 kDa) eight-arm star topologies over a wide range of salt concentrations ([Li/EO] ranging from 0 to 0.2) (error bars represent standard errors).

The salt concentration-dependent Li-ion conductivities with varying topologies for PEO-based electrolytes are shown in Figure 3. Irrespective of the polymer architecture, the addition of LiTFSI increases the ionic conductivity of the electrolytes at moderate salt concentration due simply to the increasing concentration. In this sense, [Li/EO = 0.085] appears to result in the highest conductivity for all architectures. Further increasing the salt concentration ([Li/EO > 0.085]) slightly reduces the ionic conductivity due to increased bulk viscosity arising from ion pairing and aggregation effects observed from XRD and FT-IR. Figure 3a compares the conductivity data for the same total molecular weight (20 kDa) PEO at 75 °C in the liquid state of all compositions. The ionic conductivity increases with increasing branching; PEOs with the most extreme branched cases, namely, HBG3 and HBG6 architectures, have almost 3 times higher ionic conductivity, whereas nonlinear architectures with a moderate number of arms, namely, four-arm and eight-arm stars, resulted in nearly 2 times higher ionic conductivity when compared to the linear analogue. The reason for this can be explained by two essential properties of the hyperbranched PEOs, which facilitated ionic conduction: higher branching of the PEOs results in a much lower bulk viscosity (see the bulk viscosity section below) and significantly increased the free volume in the architecture, facilitating ion transport (see the free volume section below), while its loose structure provides higher solubility of the salt and enhances ion diffusion in the polymer matrix^34,35^ (see Table S2 for the polymer compactness). Depending on their topology, polymer chains with the same monomer chemistry and same molecular weight can have both loose and compact forms. Uniformly distributed monomers in linear polymers possess a random coil structure with a rather isotropic spatial distribution. In the hyperbranched polymers, the distribution of the monomers is toward the end of the chains, resulting in looser structures when compared to the linear chains. However, in symmetric star polymers, by increasing the number of arms (functionality) and decreasing the arm length, the monomer density increases near the center of the star and creates a compact and impenetrable core region, while dangling free ends occupy more space. The closely packed region prevents the interpenetration of chains and makes the star polymers adopt a colloidal particle-like character at the core. The dominance of this core region can be tuned simply by changing the arm length and functionality. The extent of the hard sphere-like center relative to the radius of gyration (*R*_g_) of the star polymer is a measure of chain “compactness.”^36^ is used to estimate the compactness in the chains, where *l*_p_ is the persistence length (0.41 nm for PEO^37^). Table S2 displays the compactness of the polymers used in this study. We found that the polymers with a higher degree of branching and a looser topology show the highest conductivity. In other words, decreasing compactness of the nonlinear PEOs for all architectures at the same molecular weight (20 kDa) facilitated lithium-ion migration by enabling faster ion diffusion.^34,35^ (See the DLS Results section in the Supporting Information for details of polymer compactness obtained for various PEO topologies.)

We also investigated the effect of arm molecular weight in four-arm and eight-arm star structures. Figure 3b,c displays the changes in ionic conductivities with respect to different salt concentrations in four-arm and eight-arm star PEOs with varying arm lengths. Based on the data in Figure 3b, increasing the arm molecular weight slightly enhanced the ionic conduction in four-arm star PEO-based electrolytes. The reason why the ionic conductivity was lower for the four-arm PEO with shorter arm lengths could be related to the transient crosslinking effect arising from the strong interactions between the terminal OH and either the anion and cation, slowing down the ion transport.^7^ The number of chains in neat 4F5 and 4F10 PEO-based electrolytes is 4 and 2 times higher than the number of chains in neat 4F20-based electrolytes, respectively. This means that a higher number of OH terminated end groups in these samples causes higher transient crosslinking between four-arm star chains (4F5 and 4F10) and ions, ultimately reducing the ion conductivity in the samples. Additionally, we had the same observation for the eight-arm star PEO-based electrolytes (8F10 and 8F20); however, the ionic conductivity of 8F40 was lower than that of 8F20, which may be due to the higher bulk viscosity when compared to the other eight-arm star PEO architectures (explained in detail in the bulk viscosity section below).

**Figure 4:**
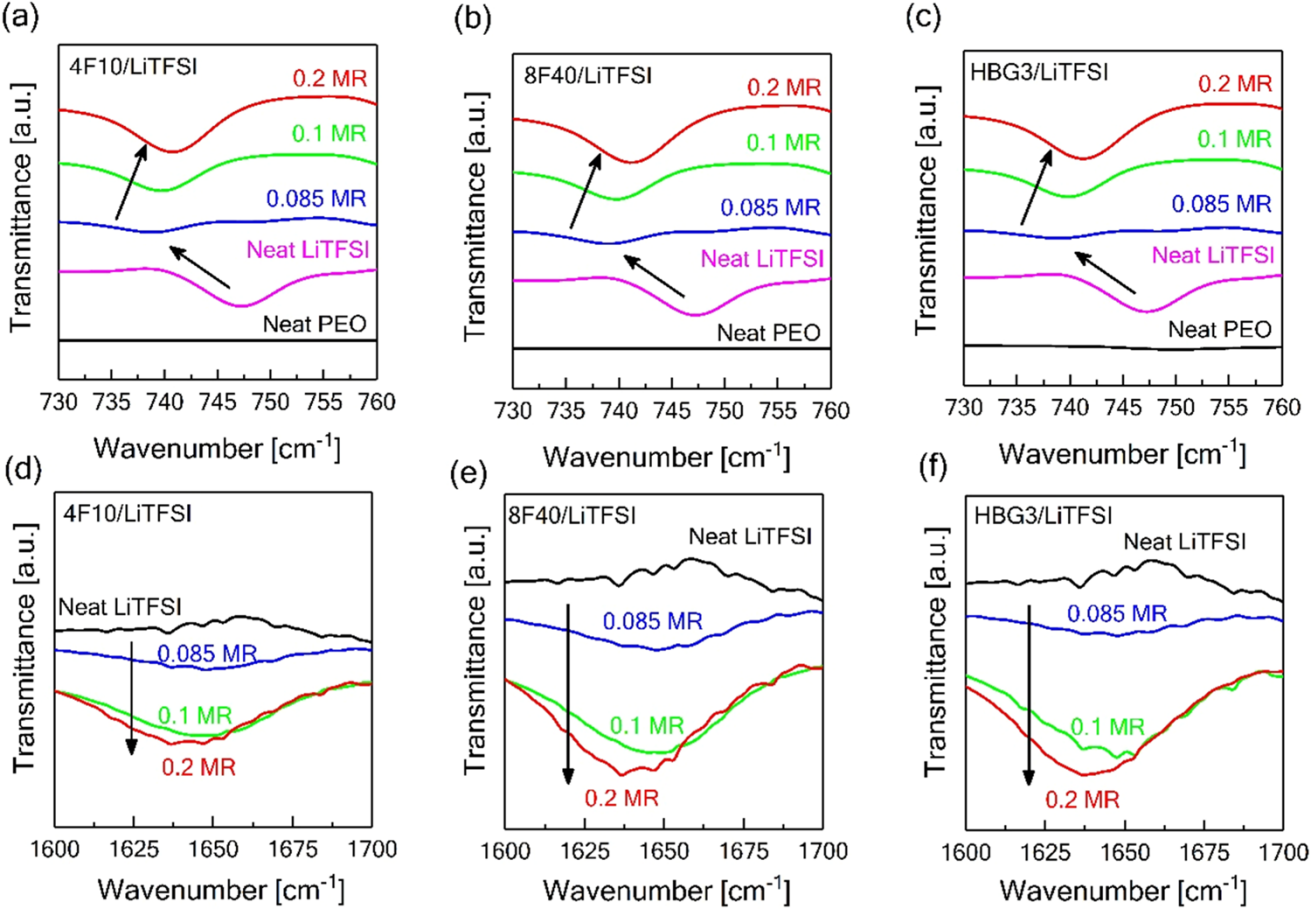
FT-IR measurements on the neat LiTFSI, PEOs, and its electrolytes containing high lithium amounts [Li/EO > 0.085] and polymer architectures of 4F10 (a, d), 8F40 (b, e), and HB3G (c, f). Data are vertically shifted for the clear observation of the changes.

**Figure 5:**
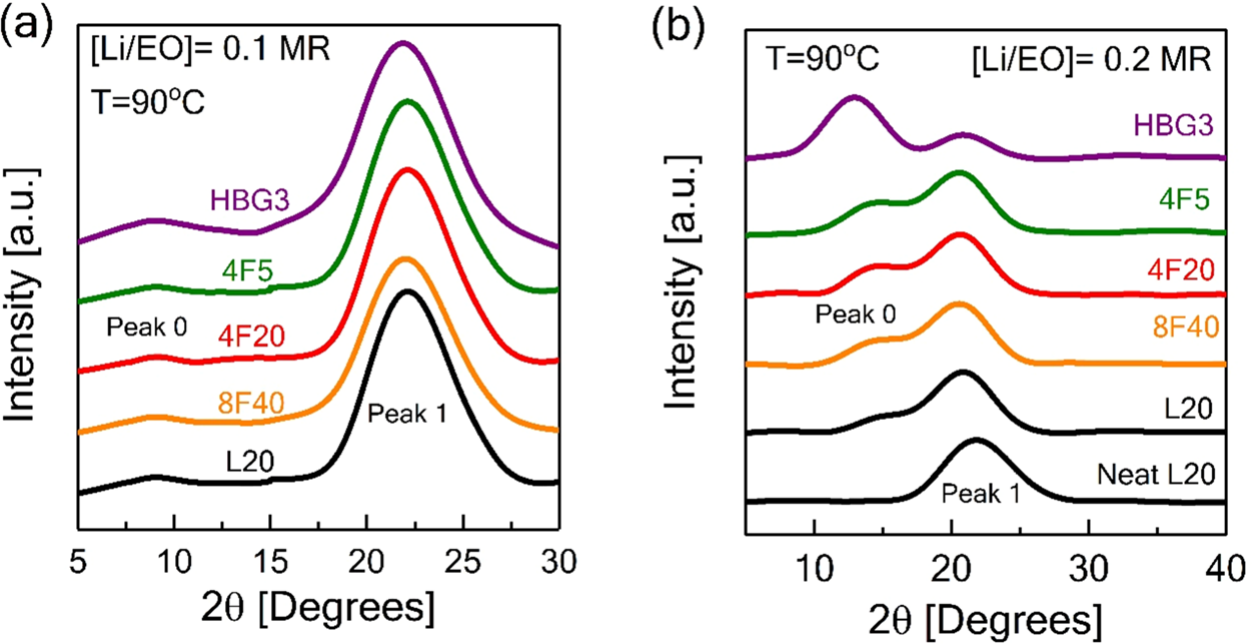
High-temperature X-ray diffraction (XRD) measurements on the neat PEO and its electrolytes with different architectures containing L20, 4F5, 4F20, 8F40, and HB3G, and high lithium concentrations (a) [Li/EO = 0.1] and (b) [Li/EO = 0.2] at 90 °C (for the XRD data, Savitzky–Golay smoothing has been applied and data vertically shifted).

The results at high salt concentrations are also important as the salt ions tend to aggregate, leading to decreased Li^+^ conductivity^38^ when the salt concentration increases above the critical point as shown by the salt concentration-dependent Li-ion conductivities in Figure 3. We performed Fourier transform infrared (FT-IR) and high-temperature XRD to investigate ion aggregation (Figures 4 and 5). Figure S3 shows the full FT-IR spectra of neat PEOs and PEO–LiTFSI salt complexes with varying salt concentrations and topologies. The characteristic peaks monitored for pure PEO at 1467, 1341, 1240, 1097, 958, and 841 cm^–1^ could be attributed to CH_2_ scissoring, asymmetric CH_2_ wagging, asymmetric CH_2_ twisting, C–O–C stretching, gauche C–C conformation, and CH_2_ rocking, respectively.^39,40^ The broad peak from 2750 to 3000 cm^–1^ corresponds to asymmetric CH_2_ stretching.^40,41^ These characteristic peaks are also seen in the case of electrolyte samples with significant changes in peak positions and intensities with increasing salt concentration for those two fundamental peaks positioned at 746 and 1634 cm^–1^. These peaks have been commonly used as representative vibrational modes attributed to ion pair formation and aggregation, respectively,^38,39,42−46^ which indicates ion pairing and clustering effects.

For the neat LiTFSI salt, the peak located at 746 cm^–1^ accounts for CF_3_ bending coupled with the S–N stretching vibration of the TFSI anions.^38,45,46^ After the dissolution of the salt in different PEO matrixes ([Li/EO] = 0.085), this peak shifted to the lower value of 738 cm^–1^ (see the left arrow in Figure 4a–c), which is associated with the coordination of Li^+^ with PEO, and forming free TFSI^–^ anions, in agreement with previous reports.^38,39,42^ When the molar ratio ([Li/EO]) increased to 0.1 and 0.2, respectively, the peak got broader and shifted to higher wavenumbers, from 738 to 743 cm^–1^ (see the right arrow in Figure 4a–c). It shows an enhancement in the coordination of Li^+^ with TFSI^–^ anions, forming ion pairs, resulting in electrolytes with lower ionic conductivity due to less free TFSI^–^ ions. Additionally, since the position of the peak at 1467 cm^–1^ remained unchanged among all FT-IR spectra for PEOs, it was used as a reference point in comparison for the intensity ratios regarding the transmittance of the peak position at 1634 cm^–1^ corresponding to LiTFSI aggregation. It was found that this peak reduced more with increasing salt content and there was a slightly higher decrease with an extreme degree of branching such as a hyperbranched polymer architecture (see the downward arrow in Figure 4d–f). This confirms the formation of LiTFSI aggregates, which could be attributed to the higher ion dissociation with increased branching.^43,47,48^

Figure 5 shows the XRD intensity profiles as a function of topological variations for the neat PEO and different salt concentrations performed at 90 °C in PEO/LiTFSI complexes. In the case of the salt-free sample for linear PEO (L20), the intensity profile only reveals one peak at the 2θ of ≈22°, attributed to the amorphous halo of the PEO chains marked as “Peak 1” in Figure 5.^9^ This peak slightly shifts to smaller 2θ in all electrolytes due to swelling of the chains with salt, which increases the interchain correlation distance. Remarkably, by increasing the salt concentration with the molar ratios ([Li/EO]) of 0.1 and 0.2 for all different architectures of PEO, another peak marked as “Peak 0” appears at a lower 2θ angle of around 9°, which becomes more intense at the highest salt content ([Li/EO = 0.2]) at around 15°. In agreement with the previous results and supporting our FT-IR results, this is associated with ion cluster formations.^9^

Furthermore, we would also like to state that the polymer architecture controls the intensity of the ionic clusters with an extremely high salt concentration ([Li/EO = 0.2]) as seen in Figure 5b. More specifically, we observed that the intensity of Peak 0 increased gradually with an increasing degree of branching and maximized significantly for the hyperbranched case. The representative calculations for the 2θ angles, scattering vector, and the distance between clusters with the corresponding polymer architecture are given in Table S3. Peak 0 generally moves to smaller *Q*-values with increasing branching, indicating that the length scales associated with the correlations between clusters slightly increase with the degree of branching. The average size of the formed clusters is estimated from the Scherrer equation *L* = *K*λ/β cos θ, where L is the crystallite size, λ = 0.154 nm is the X-ray wavelength, and *K* ≈ 1.1 is the shape factor (see Table S3 in the SI for the values). The size of the crystallites ranges from ≈6.95 nm (for 4F20) to ≈3.95 nm (for HB3G), but there is no systematic variation of size with architecture in contrast to the monotonic shift in the Peak 0 position. Considering the smallest average size observed for HG3G along with its enhanced Peak 0 intensity suggests the presence of more inhomogeneously dispersed clusters in the highly branched matrix. These results, thus, indicate that in addition to the modified phase diagrams, cluster size and distribution of salt aggregates can be modulated (at a fixed salt concentration) by controlling the spatial distribution of monomers within a single polymer chain.

### Bulk Viscosity of Electrolytes

**Figure 6:**
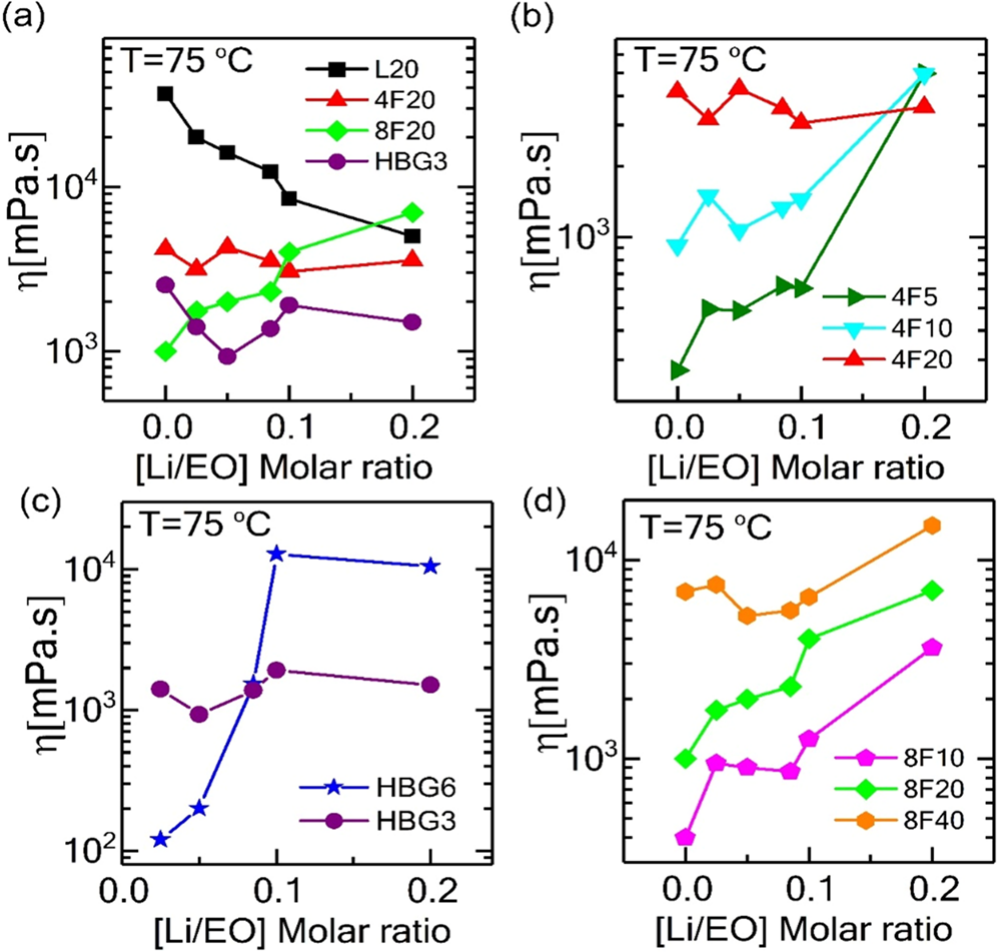
Viscosity of the PEO-based electrolytes at 75 °C with various architectures including linear, star, and hyperbranched topologies with (a) the same molecular weight (*M*_w_ ∼ 20 kDa), (c) hyperbranched architectures (b, d) with varying molecular weights (*M*_w_ ∼ 5, 10, 20 kDa) for four-arm and (*M*_w_ ∼ 10, 20, 40 kDa) eight-arm star topologies over a wide range of salt concentrations ([Li/EO] ranging from 0 to 0.2).

The conductivity of the polymer electrolyte can be explained in terms of the trade-off between an increasing number of charge carriers and ion pairing/clustering effects, which strongly impact bulk viscosity. We observed a Newtonian behavior (see Figure S4 in the SI) in the viscosity measurements of the electrolytes at 75 °C (above the melting temperature of the samples), which is due to the relatively low *M*_w_ of the polymers. Figure 6a shows the viscosity of the PEO/LiTFSI samples with four different architectures (linear, four-arm star, eight-arm star, and hyperbranched) with the same total *M*_w_ (20 kDa). The effect of salt addition on the viscosity of the samples is twofold. Ions would act as solvents in the polymer matrix and reduce the melting temperature (as seen in Figure 2) and bulk viscosity of the samples. In addition, the higher entanglement of the linear polymer leads to the higher viscosity of this sample. On the other hand, a shorter chain length in nonlinear polymers lowers the interpenetrability of the polymer chains and induces a lower viscosity (see Figure 3a). By increasing the salt content, the viscosity of the linear polymer decreases, as the ions act like solvents for the linear chains and the melting temperature decreases. Note that the formed clusters, as XRD results suggested, are smaller in size; thus, they probably did not reach a critical size to increase the bulk viscosity.^49^ Also, the number density of the end groups for the linear long-chain PEO is low; thus, the effect of possible transient crosslinking between the chains via stronger Li-OH complexation is negligible. This is different in the branched structures. The viscosity of the electrolytes with the four-arm star does not vary too much due to the balance between reduction in melting temperature and chain–ion interactions. However, as the functionality increases in eight-arm stars, the transient crosslinking between the −OH terminated end groups of the star arms via salt ions increases, which leads to higher bulk viscosity of these samples at elevated salt concentrations. Note also that the clustering is much more severe as seen in the XRD results. Figure 6b,d compares the viscosity of the samples with four-arm and eight-arm star polymers but different arm lengths. For the eight-arm star, the trend is the same for all arm lengths as these already involve a large number of chain terminals. For the lightly functionalized (four-arm) star (Figure 6b), the effect of end groups on salt-concentration-dependent viscosity is more apparent as decreasing the arm length from 5 to 1.25 kDa resulted in a behavior similar to the highly branched eight-arm stars (in Figure 6d). For hyperbranched samples (Figure 6c), viscosity decreases in low salt concentrations ([EO/Li] <0.05) as in the linear polymer case due to its linear backbone coordinating with dissolved ions; however, for [EO/Li] > 0.05 ratios, viscosity increases rapidly due to ion clustering. At the highest concentrations, the viscosity levels off due to large agglomerates (as opposed to rather dispersed clusters in stars).

### Effect of Free Volume

**Figure 7:**
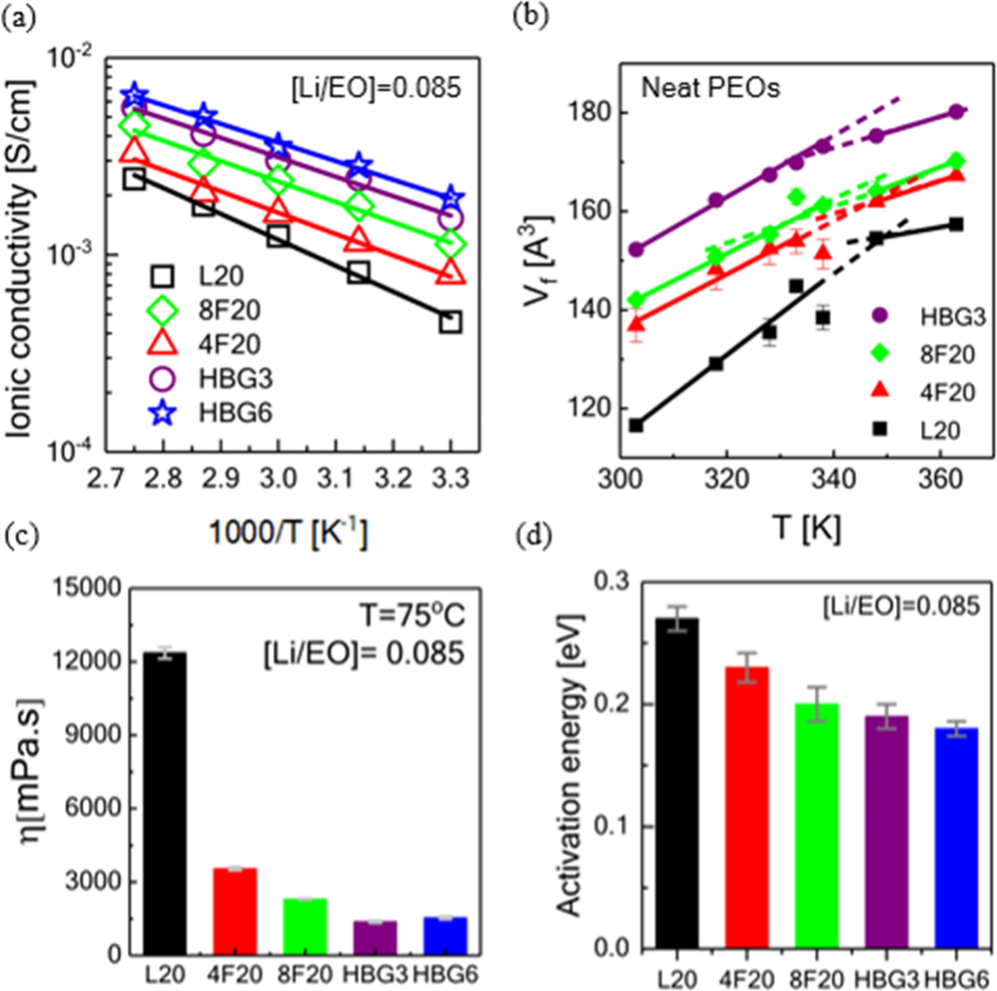
Ionic conductivity measurements of PEO-based electrolytes as a function of temperature with various architectures including linear, star, and hyperbranched topologies with (a) the same molecular weight (*M*_w_ ∼ 20 kDa) over a salt concentration [Li/EO] ranging from 0.085 to 0.1. (b) Temperature-dependent free volume measurements of neat PEOs with different topologies probed by PALS (error bars represent 1 standard deviation). (c) Zero-shear viscosity measurements of PEO/LiTFSI salt complexes with [Li/EO] = 0.085 at 75 °C. (d) Estimated pseudo-activation energies for electrolytes with different topologies.

We investigated the temperature-dependent ionic conductivity of all PEO-based electrolytes containing constant salt loading ([Li/EO] = 0.085) (where the highest conductivity is observed for all PEO architectures) between 30 and 90 °C with Δ*T* = 15 °C to gain more insights into the mechanism of ionic mobility. The results are shown in Figure 7a. We have employed both Vogel–Fulcher–Tammann (VFT) and Arrhenius fittings to the ionic conductivity data with respect to temperature. In the case of VFT fitting, *T*_v_ values were very close to 0 K (ranging between 0 and 10 K), meaning that applied fits converge to the Arrhenius mechanism. Therefore, we continued to apply Arrhenius behavior on the ionic conductivity, which suggests that the ion transport is mainly controlled and facilitated by the bulk viscosity (Figure 7c). Increased free volume (Figure 7b) in nonlinear topologies with a higher degree of branching is the primary conduction mechanism, rather than ion hopping in these polymer electrolytes^50^ (see the Positron Annihilation Lifetime Spectroscopy section in the Supporting Information for details of free volume measurements). This is also supported by the fact that these polymer electrolytes consisting of a constant salt ratio of [Li/EO] = 0.085 have insignificant differences in their glass-transition temperatures, implying that the segmental dynamics are similar (also shown directly by QENS in the next section), and it is not primarily responsible for the differences seen in the ionic conductivities. Furthermore, when the sole effect of polymer architectures having similar arm lengths such as eight arms (2.5 kDa) and linear (1.5 kDa) on the ionic conductivity was investigated, it was observed that the architectural changes did increase the ionic conductivity. The changes in the ionic conductivity of nonlinear PEO architectures are quite important to develop better polymer electrolytes (SPEs) due to two fundamental reasons, such as lower viscosity (Figure 7c) and substantially increased free volume (Figure 7b) in highly branched PEO architectures as probed by PALS. Additionally, the free volume data for linear PEO were found to be comparable to the previously reported values.^41,51−53^

More specifically, the ionic conductivity of the PEO-based electrolytes with extreme branching such as hyperbranched architectures has shown as much as a threefold increase. This can be attributed to its loose structure leading to an order of lower bulk viscosity and increased free volume (by 20%) compared to the linear counterpart. On the other hand, this increase in ion transportation of PEO electrolytes with other nonlinear topologies (eight arms and four arms) was nearly twofold, mainly due to the higher viscosity and less increased free volume (as much as 10% when compared to the linear one) in the case of star architectures. These indicate that ion transport is affected by the bulk viscosity and facilitated by the free volume in the polymer matrix. This is further supported by the activation energies estimated from the Arrhenius fits displayed in Figure 7d. The Arrhenius model is given by^13^
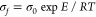
1where σ_0_ is the reference conductivity, *E* is the active energy, and *R* is the gas constant number (8.314 J·K^–1^·mol^–1^). Fitting the conductivity data to the Arrhenius model gives the estimated pseudo-activation energies as shown in Figure 7d. The activation energies are highly architecture-dependent and in parallel to the conductivity data results.^13^ The electrolyte with the linear PEO has an activation energy ranging around ≈0.27 eV for the salt molar ratio of [Li/EO] = 0.085, which results in the lowest ionic conductivity among the PEO investigated architectures. On the other hand, activation energy decreased to 0.23 and 0.22 in the case of star architectures and further lowered to 0.20 and 0.19 eV for HBG3 and HBG6, respectively, which is the highest ionic conductivity among the PEO investigated architectures. (All of these activation energies are comparable to the previously reported values.^13,54^) Eventually, our study quantitatively shows the relationship between ion conduction and two other significant and dominant factors, including free volume and bulk viscosity in neat PEO-based electrolytes. Our study clearly shows that nonlinear architectures did increase the ionic conductivity mainly due to their increased free volume with a lowered bulk viscosity. More specifically, when compared to two significant cases with similar arm lengths including eight-arm star (20 kDa) and linear topology (1.5 kDa), we can conclude that nonlinear topologies could be used to enhance the ionic conductivity by just modifying the architecture. It is very important to mention that as a result of decreasing the arm length in linear PEO, ionic conduction could be increased due to facilitated movements of the ions provided by the lowered viscosity. However, this increase in ionic conduction is further enhanced when nonlinear architectures are applied, which could pave the way for the employment of branched architectures in polymer electrolytes.

### Polymer Segmental Dynamics in Electrolytes

The addition of salt is known to decrease segmental dynamics of amorphous linear PEO, which can be directly measured by quasielastic neutron scattering (QENS).^16,55^ Balsara and co-workers studied a range of salt concentrations ([Li/EO]) from 0 to 0.2 and showed an exponential increase in the monomeric friction coefficient, ζ, with salt concentration. The ionic conductivity was directly related to the change in segmental dynamics of linear PEO; yet, whether the effect of salt addition on the segmental dynamics of PEO differs in different architectures is yet to be discovered. We performed QENS experiments on salt-free neat PEO and PEO electrolytes containing a fixed salt concentration yielding [Li/EO] = 0.08 at 400 K above the melting temperatures. In QENS, the self-motion of H-atoms at the nanoscale time scale is due to the translational motion of the segments along the polymer backbone that is captured at a sufficiently low scattering vector relevant to the monomer length scale (≈1 nm). The simultaneous access to the monomeric length scale and associated relaxation spectra thus allows direct measurement of the polymer segmental relaxation rate even in the presence of a salt.

**Figure 8:**
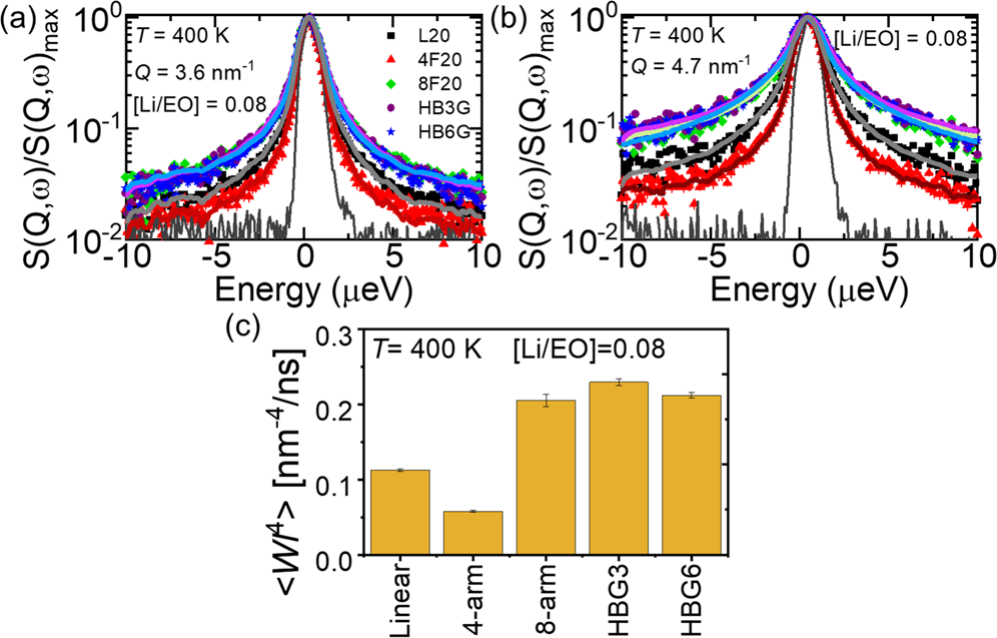
Normalized dynamic structure factors obtained for PEO electrolytes with different architectures at constant [Li]/[EO] = 0.08 and at (a) *Q* = 3.6 nm^–1^ and (b) *Q* = 4.7 nm^–1^. The measurements were performed at 400 K. The lines are fit to a Fourier transform KWW function (see the text for details). (c) Average elementary Rouse relaxation rates, ⟨*Wl*^4^⟩, estimated from fitting the S(*Q*, ω) for *Q* = 3.6 nm^–1^ and *Q* = 4.7 nm^–1^. The values are given in Table S4.

Figure 8a,b compares the normalized dynamic structure factors (S(*Q*, ω)/S(*Q*, ω)_max_) obtained at *Q* = 3.6 nm^–1^ and at *Q* = 4.7 nm^–1^, respectively, for the PEO-LiTFSI electrolytes with 20 kDa total *M*_w_ and at the fixed salt concentration [Li/EO] of 0.08. The sharp solid line is the spectrum from a fully elastic vanadium sample representing the instrument resolution. The nanoscale motion of H-atoms on the PEO backbone results in energy exchange between the neutrons and the sample, leading to quasielastic broadening with respect to the resolution. The width of the peaks is inversely related to the underlying relaxation time (in this case, segmental relaxation time). Even from the raw data, it is clearly seen that the segmental dynamics in PEO electrolytes are different and nonmonotonic in the nonlinear polymer architectures. The four-arm star, which is lightly branched and compact, exhibits a slower segmental relaxation compared to the linear chain due to its low number of free ends insufficient to overcome the dynamic slowdown near the densely packed star core. The highly branched polymers, including eight-arm star, HBG3, and HBG6, display remarkably enhanced elementary Rouse relaxation rates compared to the linear one. However, the spectra of these polymers are almost indistinguishable; the overall segmental dynamics in this set of branched polymers is practically architecture-independent.

To compare more quantitatively, we fitted the dynamic structure factors, S(*Q*, ω), to the Fourier transform KWW function (which is essentially a stretched exponential function, exp[−(*t*/τ)^β^]) convoluted with the instrument resolution. Here, β is the stretching exponent and τ is the relaxation time. A delta function and a constant background were also added to account for possible residual immobile fractions and very fast localized motions.^56,57^ The fitted curves are shown as solid lines on the spectra (note that the elementary Rouse rates for the neat polymers except for HBG6 were reported in our previous work^56^). It was found that β is very close to 0.5 for all samples, confirming that dynamics is due to the curvilinear multimodal motion of the segments along the backbone (i.e., Rouse motion). The *Q*-dependent relaxation time is, thus, related to the elementary Rouse relaxation (segmental relaxation) parameter (*Wl*^4^) by *Wl*^4^ = 9π/(*Q*^4^τ), where *l* is the segment length (≈0.58 nm for PEO) and *W* = 3*k*_B_*T*/ζ*l*^2^ is the relaxation rate. Figure 8c shows the ⟨*Wl*^4^⟩ averaged from the data at *Q* = 3.6 nm^–1^ and *Q* = 4.7 nm^–1^ (which are very close and have the same architecture dependence; see Figure S6). The values are also listed in Table S4. The nonlinear polymer architectures, except for the four-arm star, exhibit practically indistinguishable segmental relaxation at the same salt concentration despite the large variation in ionic conductivity as shown in Figure 7a. For the four-arm star PEO electrolyte, the segmental dynamics is even lower (by about half) compared to the linear PEO, whereas its ionic conductivity was considerably higher. Our results, therefore, provide direct experimental evidence of the unusual decoupling of the ionic conductivity from the segmental dynamics in these architecturally engineered polymer electrolyte samples. The QENS results verify that the ionic conductivity is mainly controlled by free volume increase in samples due to branching. These results call for new theoretical and experimental studies on the complex interplay between the macromolecular architecture and ionic conductivity for the rational design of solvent-free flexible batteries.

## Conclusions

The series of different topologies (linear, star, and hyperbranched) for PEO polymers with varying concentrations have been thoroughly investigated to correlate the phase diagram, free volume, bulk viscosity, and segmental dynamics with ionic conductivity in PEO/LiTFSI electrolytes. The constructed phase diagrams, which depend on the employed PEO architecture and salt concentration, showed an effectively decreasing degree of crystallization with increasing branching and salt addition owing to the restricted mobility of the polymer chains. The completely amorphous electrolytes were obtained using lesser salt concentration with a higher degree of branching, especially significantly lower in the case of hyperbranched PEO architectures. Furthermore, the salt concentration-dependent Li-ion conductivities for different PEO topologies unveiled the ionic conduction for all architectures maximized with the molar ratio of [Li/EO = 0.085], above which it decreased drastically due to the formation of ionic clustering suggested by XRD. The PEO electrolytes including highly (hyperbranched) and moderately branched architectures (four arms and eight arms) with the molar ratio of [Li/EO = 0.085] resulted in nearly threefold and twofold higher ionic conductivity at 60 °C, respectively, when compared to the linear analogue, which is attributed to the enhanced free volume in nonlinear topologies due to unconnected chain ends. In addition, regardless of the PEO architectures, the temperature dependence of ionic conductivity in neat PEO/LiTFSI electrolytes with a fixed salt content ([Li/EO = 0.085]) was well defined using the Arrhenius mechanism, suggesting that ion transport is essentially affected by bulk viscosity and free volume. The QENS results also show that the average segmental dynamics in the highly branched electrolytes are faster than linear PEO, yet there is no significant variation between different architectures. Thus, our results suggest that the coupling of ionic conductivity from segmental dynamics is not as strong in the nonlinear architectures, which offers significant potential for developing new mechanically strong yet highly conductive electrolytes based on the macromolecular architecture.

## Supporting Information Available

X-ray diffraction (XRD); differential scanning calorimetry (DSC); and electrochemical impedance spectroscopy (EIS) results (PDF)
