# Supplementary material for: Decoding Polymer Architecture Effect on Ion Clustering, Chain Dynamics, and Ionic Conductivity in Polymer Electrolytes
Source: ACS Appl Energy Mater. 2023 Mar 23;6(7):4053–64. doi: 10.1021/acsaem.3c00310 (PMC10091352; doi:10.1021/acsaem.3c00310)
Supplement: Supplementary file 1 — ae3c00310_si_001.pdf [file ae3c00310_si_001.pdf]

Supporting Information

Decoding Polymer Architecture Effect on Ion-Clustering, Chain Dynamics and Ionic Conductivity in Polymer Electrolytes

Recep Bakar,[1,¶] Saeid Darvishi,[2] Umut Aydemir,[3,4] Ugur Yahsi,[5] Cumali Tav,[5] Yusuf Ziya Menceloglu,[6] Erkan Senses[2,4,7*]

[1]*Department of Material Science and Engineering, Koç University, Sariyer, Istanbul 34450, Türkiye*

[2]*Department of Chemical and Biological Engineering, Koç University, Sariyer, Istanbul 34450, Türkiye*

[3]*Department of Chemistry, Koc University, Sariyer, Istanbul 34450, Türkiye*

[4]*Koc University Boron and Advanced Materials Application and Research Center (KUBAM), Sariyer, Istanbul 34450, Türkiye*

[5]*Department of Physics, Faculty of Science, Marmara University, Kadikoy, Istanbul 34722, Türkiye*

[6]*Faculty of Engineering and Natural Sciences, Sabanci University, Tuzla, 34956, Istanbul, Türkiye*

[7]*Koç University Surface Science and Technology Center (KUYTAM), Rumelifeneri yolu, 34450, Sariyer, Istanbul, Türkiye*

¶*Present address: Department of Cell R&D Engineering, Siro Silk Road Clean Energy Solutions, Gebze, Kocaeli 41400, Türkiye*

**Corresponding author:* esenses@ku.edu.tr

EXPERIMENTAL METHODOLOGY

Materials

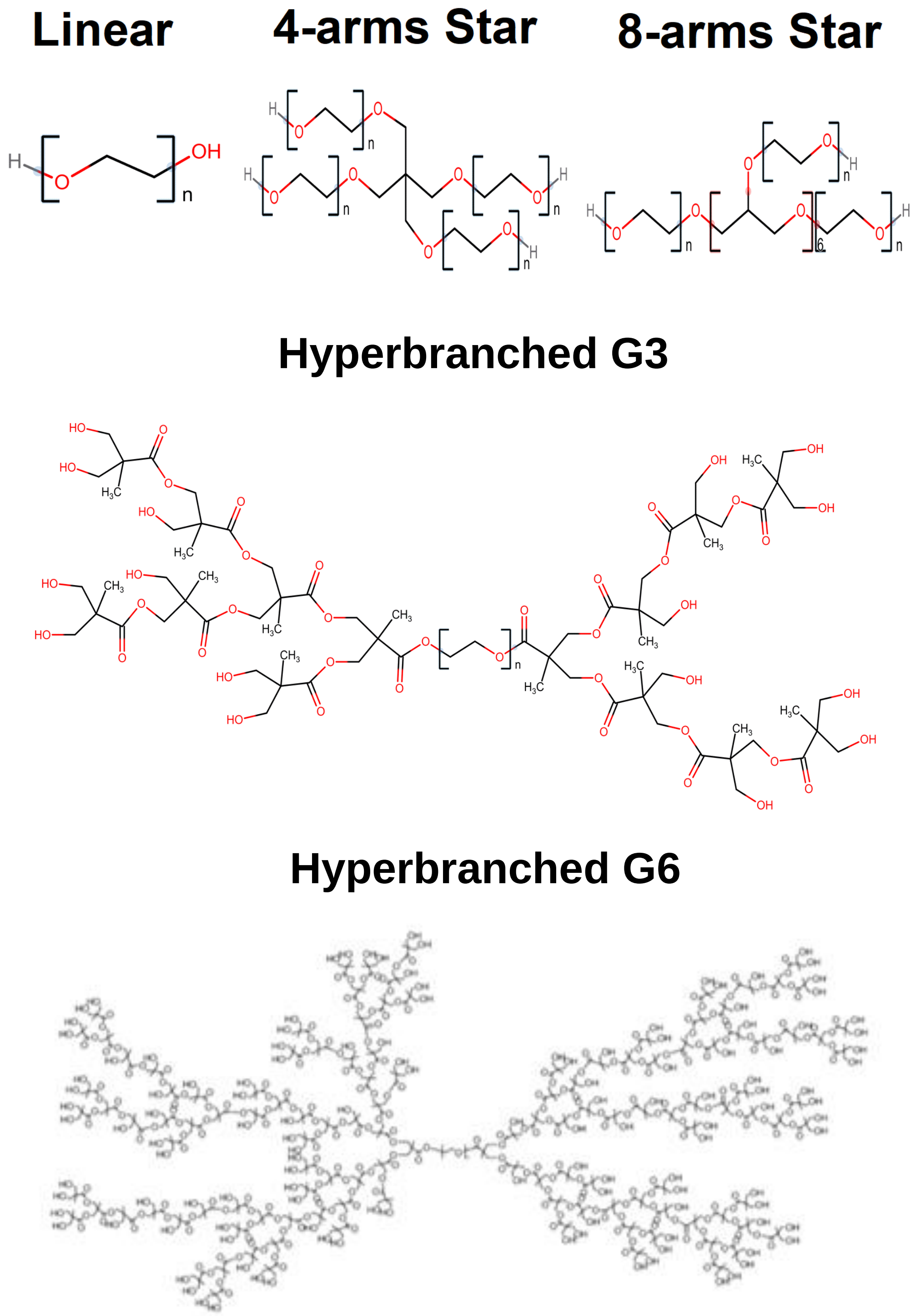

Figure S1. Chemical structure of the PEO polymers. The structure for the hyperbranched PEO (G6) was taken from the manufacturer (Sigma-Aldrich)'s page.

RESULTS AND DISCUSSION

Differential Scanning Calorimetry (DSC):

To estimate the degree of crystallinity for neat PEOs with the different number of arms and arm molecular weights, we used the following equation $X_c = \left(\frac{\Delta H_m}{\Delta H_c}\right) * 100$, where ΔH_m is the melting enthalpy of the sample estimated from DSC (see the experimental results in Table S1 in the Supporting Information), and ΔH_c is the melting enthalpy of completely crystalline PEO, which is 196.4 J/g.[1]

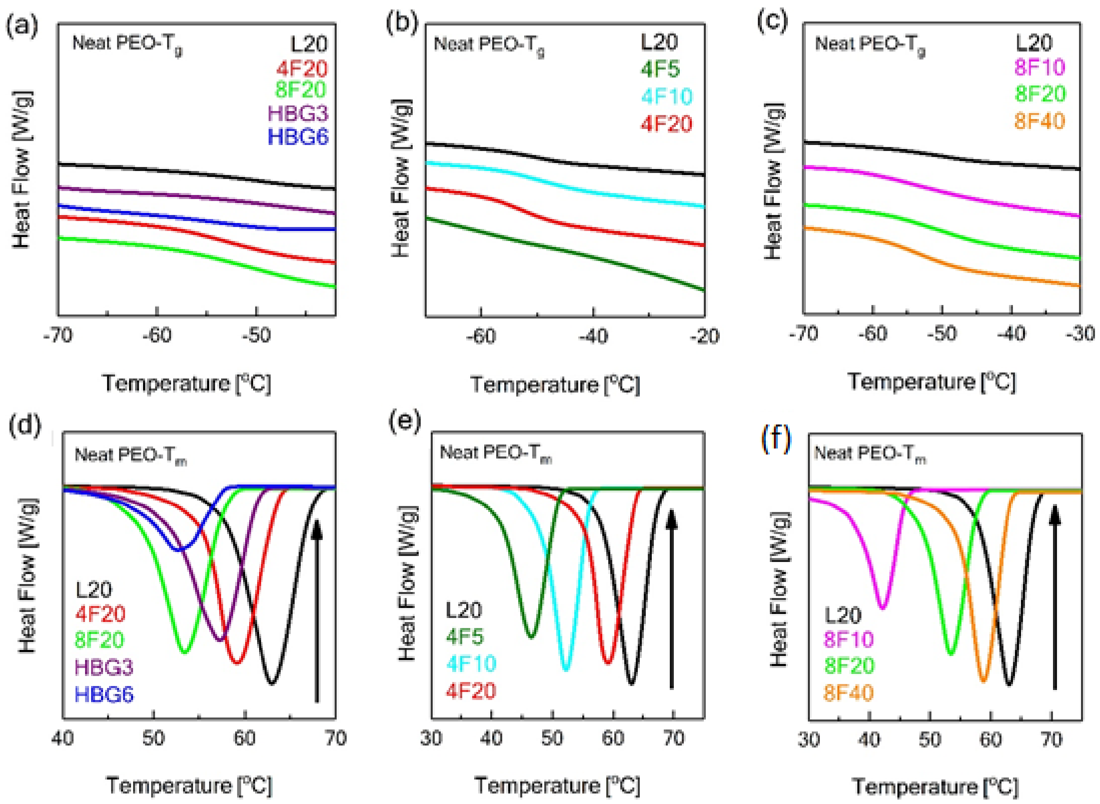

Figure S2. DSC thermographs with emphasis on glass transition (a,b,c) and melting (d,e,f) temperatures for the neat PEOs with various architectures (Exothermic peaks are shown with the arrows on the figure).

Table S1. Glass transition and melting temperatures with the estimated enthalpies and degree of crystallizations of various PEO architectures over different salt fraction.

Estimated Parameters	PEO topology with varying molecular weights									
	Li/EO	L20	4F5	4F10	4F20	8F10	8F20	8F40	HBG3	HBG6
T (Melting Temp),C	0	63	46.5	52.2	59.1	42.2	53.4	58.8	57.3	52.7
	0.025	56.9	42.1	45	51.4	38.62	44.2	52.6	49.1	44.1
	0.05	50	34.4	30.5	42.7	36.77	33	39.3	42.53	-
	0.085	30.27	-	-	-	-	-	-	-	-
Tg (Glass Transition),C	0	-51.06	-58.5	-50.7	-52.5	-53	-50.8	-53.9	-47.18	-48.4
	0.025	-45	-46.81	-49	-43.2	-51.74	-49.6	-43.4	-46.69	-45
	0.05	-41	-43.25	-53.43	-42.44	-49	-47.8	-46.2	-50	-41.2
	0.085	-46	-48	-48	-41	-45	-46.99	-46.4	-46.6	-41.4
	0.1	-43	-44.1	-44.5	-40.91	-46.77	-44.5	-44.34	-45.8	-40.3
	0.2	-38	-31	-33	-33.62	-50	-43.2	-41.32	-54	-39.8
Melting Enthalphy J/g	0	146.8	117.5	122.57	120	79.383	126.13	135.3	122.5	61.7
	0.025	86	68	70	74.2	53.614	103.58	74.7	58.7	2.4
	0.05	35.4	33	42.4	48.5	6.43	46	35.6	29.507	-
	0.085	7.51	-	-	-	-	-	-	-	-
The degree of crystallazation , %	0	74.9	60.499	62.4084	61.2	40.5	64.4	69	62.5	31.5
	0.025	43.9	34.7	35.6415	37.8	27.3541	52.8	38.1	28.1	1.2
	0.05	18.1	16.8	21.6	24.7	3.28061	23.5	18.2	15.0546	-
	0.085	3.8	-	-	-	-	-	-	-	-

Fourier transform infrared Spectroscopy (FT-IR):

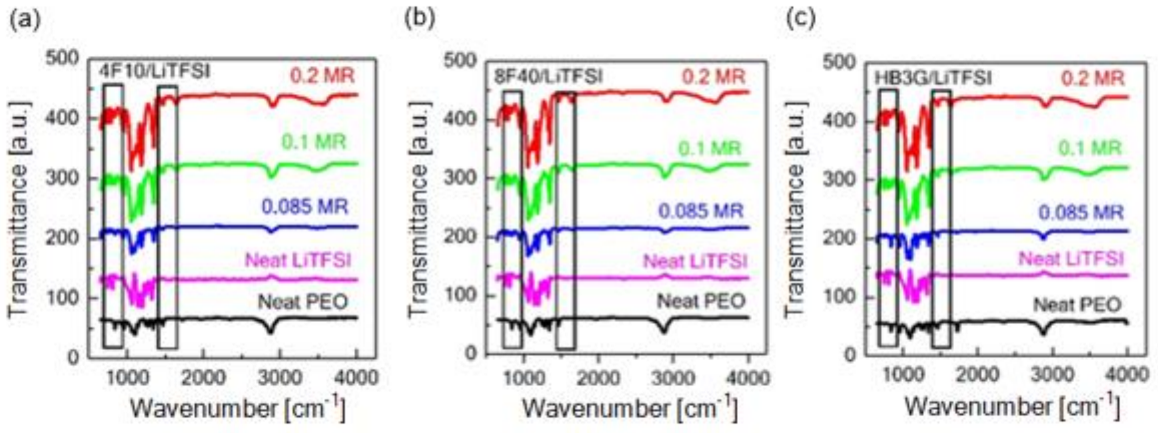

Figure S3. Full spectra of the FT-IR measurements on the neat LiTFSI, PEOs and its electrolytes containing different lithium amounts [Li/EO>0.085] and polymer architectures of 4F10 (a), 8F40 (b), HB3G (c), respectively.

X-ray Diffraction (XRD):

Table S2. The representative calculations for the 2Θ angles, scattering vector, and the distance between clusters with the corresponding polymer architectures. K≈1.1 is chosen as a constant shape factor.

Salt Molar Ratio	PEO Architecture	2Θ[°]	Q=4πSinΘ/λ [1/nm]	β [°]	D=Kλ/(β.cosΘ) [nm]
[EO/Li]=0.2	HB3G	11.5	8.1	2.47	3.95
	4F5	12	8.5	1.87	5.26
	4F20	12.25	8.7	1.40	6.95
	8F40	12.5	9.6	1.85	5.20
	L20	14	9.9	1.89	5.15

Rheology Measurements:

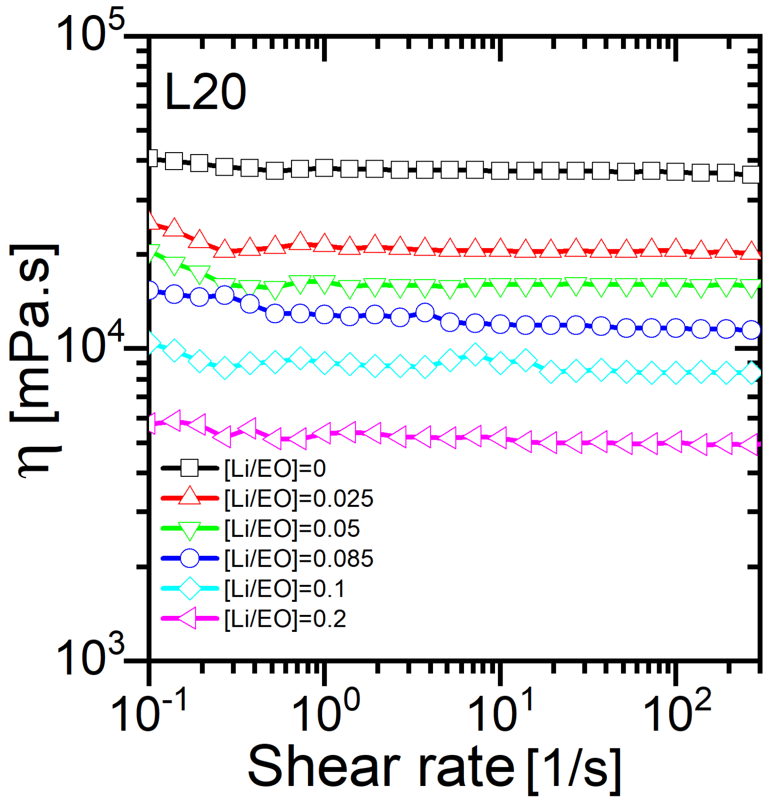

Figure S4. Viscosity of L20 electrolytes with respect to lithium content (0 < [Li/EO] < 0.2) show Newtonian flow behavior at 75°C

Dynamic Light Scattering (DLS):

Table S3. The change of compactness with respect to different polymer architectures.

PEO	M_{total} (kDa)	M_{arm} (kDa)	PID	R_g (nm)	R_h (nm)	R_{core}/R_g
**8F10**	10	1.25	1.02	2.02	1.43	0.27
**4F5**	5	1.25	1.03	1.93	1.70	0.21
**8F20**	20	2.5	1.10	2.90	2.28	0.13
**4F10**	10	2.5	1.03	2.72	2.04	0.11
**8F40**	40	5	1.09	4.04	3.07	0.07
**4F20**	20	5	1.03	3.85	2.41	0.05
**L20**	20	10	1.5	5.2	3.22	≈ 0
**HBG3**	20	-	<1.5	-	2.94	≈ 0

Positron annihilation lifetime spectroscopy (PALS): Positron annihilation lifetime spectroscopy (PALS) is a well-established and very sensitive non-destructive spectroscopy technique that allows studying a variety of phenomena and material properties on an atomic scale including free volume and its fraction.[2-5] The PALS spectra were fitted using the computer program LT polymers into four lifetime components corresponding to annihilation of para-positronium (p-Ps), free positrons, and two ortho-positronium (o-Ps) in the increasing order of their lifetime, respectively: τ_1 (the shortest-lived component with an intensity I_1), τ_2 (the intermediate-lived component with an intensity I_2), and τ_3 and τ_4 (the longest-lived components with intensities I_3 and I_4). [2-5] τ_1 and I_1 are associated with the annihilation of para-positronium (p-Ps), τ_2 and I_2 are allied with direct annihilation of positrons, and τ_3 with I_3 and τ_4 with I_4 are associated with pick off annihilation of ortho-positronium (o-Ps within the

crystalline and amorphous regions of the polymers. In estimating the longest lifetime parameters, τ_1 and τ_2 were assumed as independent of free volume and the former was assumed not to chance in vacuum and matter so we have taken as 125 ns fixed. O-Ps lifetimes, τ_3 and τ_4, are sensitive to the free volume hole size and I_3 and I_4 have straight correlation with the number of free holes in the materials. [2, 5, 6]

The relation between o-Ps lifetime and radius of free volume hole (R) is given by Tao-Eldrup model with the assumption of spherical holes as follows:[3-5, 7]

$$\tau(ns) = \frac{1}{2}\left(1 - \frac{R}{R_0} + \frac{1}{2\pi}\sin\frac{2\pi R}{R_0}\right)^{-1} \quad (1)$$

where $R_0 = R + \Delta R$ with $\Delta R = 0.1656$ nm as an empirical parameter for a measure of electron layer inside the spherical potential well. The size of free volume holes (V_f) is finally then calculated by using the following equation.

$$V_f = \frac{4}{3}\pi R^3 \quad (2)$$

The obtained results from PALS measurements for the free volume estimations with intensities I_3 and I_4 as a function of temperature and polymer topology corresponding to crystalline and amorphous regions in the polymer along with heat flow thermographs of neat PEOs with architectural changes were given by Fig S5. It is evident that there were two different clear trends for the free volume above and below a certain temperature which turns out to be melting temperature for all architectures and this temperature agrees well with the estimations from the differential scanning calorimetry experiments. This changing trend could be well related to the melting crystals in the PEO matrix, creating additional free volume significantly. This is also supported by the decreasing intensity of I_3 accompanied by the increasing intensity for I_4 (Figures S5a and S5b)

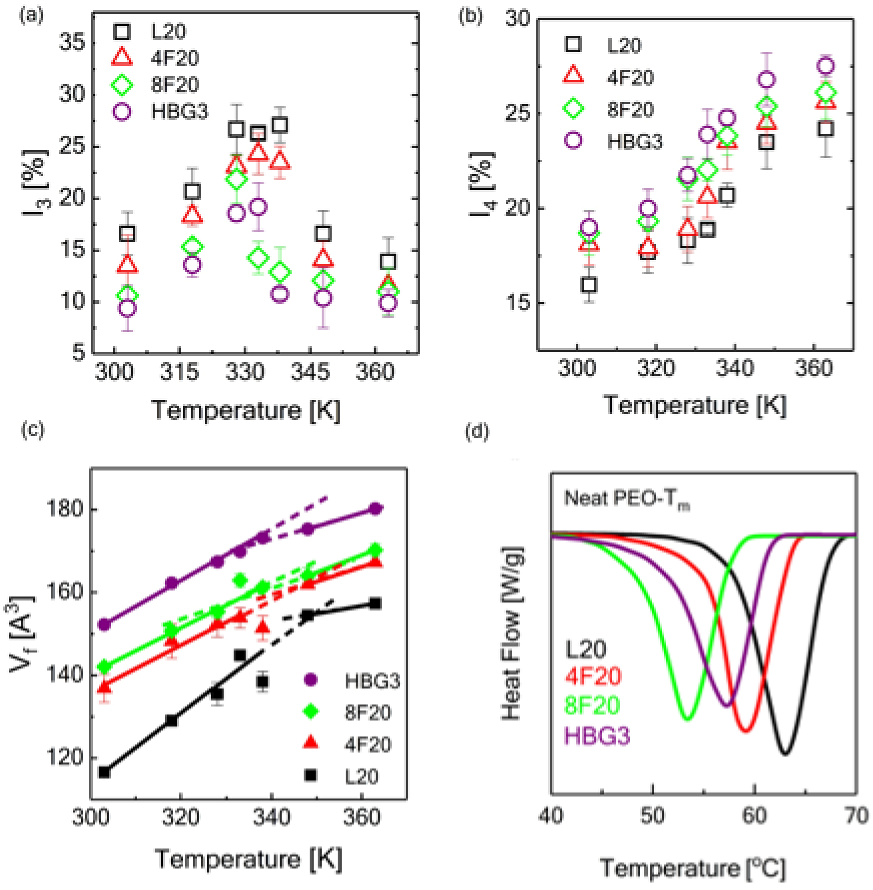

Figure S5. The intensities (a) I_3 and (b) I_4 as a function of temperature and polymer topology corresponding to crystalline and amorphous regions in the polymer, respectively. (c) Free volume measurements as a function of temperature, (d) Heat flow thermographs of neat PEOs with architectural changes.

Quasielastic neutron scattering:

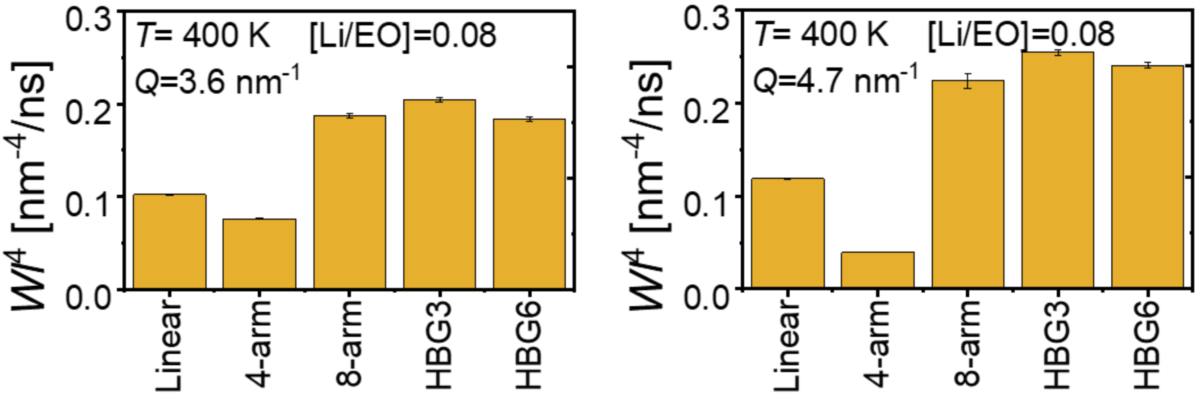

Figure S6. Elementary Rouse parameters obtained from the fittings to the S(Q,ω) data of the electrolytes at Q=3.6 nm^{-1} and Q=4.7 nm^{-1} at 400 K.

Table S4. Elementary Rouse parameters obtained from the fittings to the S(Q,ω) data from the electrolytes. The relaxation times obtained from fittings to S(Q=3.6 nm^{-1} ,ω) and S(Q=4.7 nm^{-1} ,ω) and the average Wl^4 and ζ values are given.

**PEO architecture**	**<Wl^4>** **[nm^4/ns]**	**ζ (x10^{-7})** **[g/s]**
Linear	0.110 ± 0.002	0.471 ± 0.007
4-arm	0.058 ± 0.001	0.89791 ± 0.002
8-arm	0.206 ± 0.008	0.25284 ± 0.130
Hyperbranched 3G	0.230 ± 0.004	0.22625 ± 0.083
Hyperbranched 6G	0.212 ± 0.004	0.24496 ± 0.064

References

1. Bakar, R.; Darvishi, S.; Li, T.; Han, M.; Aydemir, U.; Nizamoglu, S.; Hong, K.; Senses, E., Effect of Polymer Topology on Microstructure, Segmental Dynamics, and Ionic Conductivity in PEO/PMMA-Based Solid Polymer Electrolytes. *ACS Applied Polymer Materials* **2022,** *4* (1), 179-190.
2. Soykan, U.; Ozturk Sen, B.; Cetin, S.; Yahsi, U.; Tav, C., A detailed survey for determination of the grafted semifluorinated acrylic compound effect on thermal, microstructural, free volume, mechanical and morphological features of HDPE. *Journal of Fluorine Chemistry* **2020,** *233*, 109511.
3. Rhim, J.-W.; Kuzeci, S.; Roy, S.; Akti, N.; Tav, C.; Yahsi, U., Effect of Free Volume on Curcumin Release from Various Polymer-Based Composite Films Analyzed Using Positron Annihilation Lifetime Spectroscopy. *Materials* **2021,** *14* (19), 5679.
4. Tao, S. J., Positronium Annihilation in Molecular Substances. *The Journal of Chemical Physics* **1972,** *56* (11), 5499-5510.
5. Eldrup, M.; Lightbody, D.; Sherwood, J. N., The temperature dependence of positron lifetimes in solid pivalic acid. *Chemical Physics* **1981,** *63* (1), 51-58.
6. Yahsi, U.; Deligöz, H.; Tav, C.; Ulutaş, K.; Değer, D.; Yılmaztürk, S.; Erdemci, G.; Coşkun, B.; Yılmazoğlu, M.; Yakut, Ş., Ionic conductivity of PVdF-co-HFP/LiClO4 in terms of free volume defects probed by positron annihilation lifetime spectroscopy. *Radiation Effects and Defects in Solids* **2019,** *174* (3-4), 214-228.
7. Utpalla, P.; Sharma, S. K.; Sudarshan, K.; Deshpande, S. K.; Sahu, M.; Pujari, P. K., Investigating the Correlation of Segmental Dynamics, Free Volume Characteristics, and Ionic Conductivity in Poly(ethylene oxide)-Based Electrolyte: A Broadband Dielectric and Positron Annihilation Spectroscopy Study. *The Journal of Physical Chemistry C* **2020,** *124* (8), 4489-4501.
